# Mechanical Performance of Asphalt Materials Under Salt Erosion Environments: A Literature Review

**DOI:** 10.3390/polym17081078

**Published:** 2025-04-16

**Authors:** Wensheng Wang, Qingyu Zhang, Jiaxiang Liang, Yongchun Cheng, Weidong Jin

**Affiliations:** College of Transportation, Jilin University, Changchun 130025, China; wangws@jlu.edu.cn (W.W.); qingyuz24@mails.jlu.edu.cn (Q.Z.); chengyc@jlu.edu.cn (Y.C.)

**Keywords:** pavement performance degradation, pore characteristic, salt categories, erosion modes, chemical composition, microscopic morphology

## Abstract

Asphalt pavements are subjected to both repeated vehicle loads and erosive deterioration from complicated environments in service. Salt erosion exerts a serious negative impact on the service performance of asphalt pavements in salt-rich areas such as seasonal frozen areas with snow melting and deicing, coastal areas, and saline soils areas. In recent years, the performance evolution of asphalt materials under salt erosion environments has been widely investigated. However, there is a lack of a systematic summary of salt erosion damage for asphalt materials from a multi-scale perspective. The objective in this paper is to review the performance evolution and the damage mechanism of asphalt mixtures and binders under salt erosion environments from a multi-scale perspective. The salt erosion damage and damage mechanism of asphalt mixtures is discussed. The influence of salt categories and erosion modes on the asphalt binder is classified. The salt erosion resistance of different asphalt binders is determined. In addition, the application of microscopic test methods to investigate the salt damage mechanism of asphalt binders is generalized. This review finds that the pavement performance of asphalt mixtures decreased significantly after salt erosion. A good explanation for the salt erosion mechanism of asphalt mixtures can be provided from the perspective of pores, interface adhesion, and asphalt mortar. Salt categories and erosion modes exerted great influences on the rheological performance of asphalt binders. The performance of different asphalt binders showed a remarkable diversity under salt erosion environments. In addition, the evolution of the chemical composition and microscopic morphology of asphalt binders under salt erosion environments can be well characterized by Fourier Infrared Spectroscopy (FTIR), Gel Permeation Chromatography (GPC), and microscopic tests. Finally, the major focus of future research and the challenges that may be encountered are discussed. From this literature review, pore expansion mechanisms differ fundamentally between conventional and salt storage asphalt mixtures. Sulfate ions exhibit stronger erosive effects than chlorides due to their chemical reactivity with asphalt components. Molecular-scale analyses confirm that salt solutions accelerate asphalt aging through light-component depletion and heavy-component accumulation. These collective findings from prior studies establish critical theoretical foundations for designing durable pavements in saline environments.

## 1. Introduction

Rainfall, sea fog, and air in coastal areas contain abundant corrosive sea salt particles. The coupling effect of sea salt particles with the climate has formed a sea salt erosion environment. With the increasing requirement for transportation infrastructure, the coastal highway system is gradually being completed; hence, it is urgent to figure out the erosion mechanism of sea salt on asphalt pavement in coastal areas. In addition, a considerable amount of asphalt pavement is located in saline soils and salt lake areas in western China, such as National Highway 315 and the Qarhan Salt Lake area in China. Saline soil can result in premature cracking and fatigue damage of asphalt pavements. Meanwhile, in the saline soil and salt lake areas, asphalt pavements are exposed to salt erosion environments all year round due to the existence of salt particles in sandstorms. In northern China, snow-covered pavements are always formed due to the cold and snowy winter. It is necessary to carry out artificial snow removal to avoid traffic congestion and ensure driving safety. Currently, the main methods to deal with snow-covered pavements involve the utilization of deicing salts and the construction of self-melting snow asphalt pavements. However, these methods provide the ability and possibility for the coupling effect of salt and freeze–thaw cycles. Subsequently, fatigue cracking can be prematurely generated under the combined effect of salt and freeze–thaw, which increases the maintenance costs of the road. In summary, the serviceability and durability of asphalt pavements can be dramatically degraded in salt erosion environments. With the increasing service time of asphalt pavements, performance deterioration and durability concerns regarding asphalt pavements in salt-rich areas are more prominent, and it is imperative to clarify the interaction mechanism between salt and asphalt.

**Figure 1:**
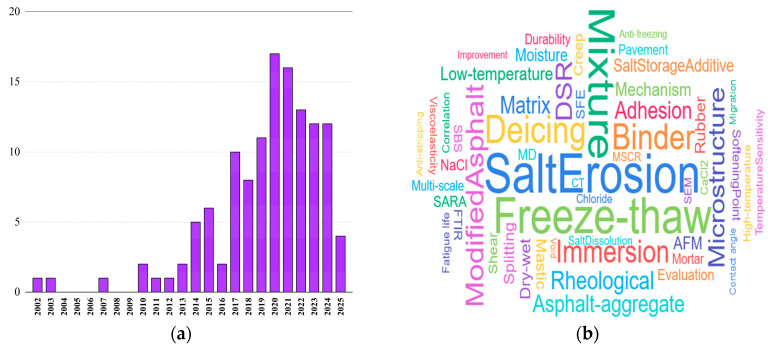
Statistics on the article quantity and keywords: (**a**) variation in the articles’ quantity with year and (**b**) word cloud of keywords.

Over recent years, extensive research has been conducted on the performance of asphalt materials under salt erosion environments. The variation in the articles’ quantity with year and the word cloud of keywords are described in Figure 1 (as of March 2025). It can be seen from the results that in recent years, there has been a significant increase in research that analyzes the influences of salt erosion environments on asphalt material performance. In addition, freeze–thaw, microscopic properties, rheological properties, etc., have become mainstream research directions. Zhang et al. [1] pointed out that salt erosion drove an increase in the heavy component content, resulting in the hardening of the asphalt binder. Moreover, salt ions can penetrate the binder, thus reducing its fatigue life. Busang et al. [2] subjected the matrix asphalt binder to immersion erosion in sodium bicarbonate (NaHCO_3_) solution, and found that the shear strain and non-recoverable creep compliance of the asphalt binder increased with the increasing saline concentration. Hence, it can be seen that the performance of the asphalt binder can be dramatically deteriorated by salt erosion. As an important component of asphalt mixtures, the performance deterioration of asphalt mortar can degrade the strength and durability of asphalt mixtures [3]. However, salt erosion environments exerted a significantly negative influence on the viscoelastic properties of asphalt mortar [4]. In addition, Han et al. [5] subjected different modified asphalt mortars to salt freeze–thaw cycles, and demonstrated that salt freeze–thaw cycles dramatically deteriorate the compressive strength of asphalt mortar. For asphalt mixtures, Wu et al. [6] analyzed the influence of salt erosion environments on the pores of asphalt mixtures containing salt storage additives, and concluded that the size and number of pores in asphalt mixtures increased with the salt erosion times. Xiao et al. [7] discovered that the adverse effect of acetic acid deicing salts on the asphalt–aggregate interface was stronger than that of pure water, and the effect enhanced with the increase in saline concentration. Wu et al. [8]. conducted freeze–thaw splitting fatigue tests on asphalt mixtures subjected to chloride salt erosion and showed that the fatigue life of asphalt mixtures was considerably diminished after chloride salt erosion. It can be seen that serious damage is generated by salt erosion on asphalt, asphalt mortar, and asphalt mixtures, which can lead to premature fatigue and cracking for asphalt pavement, thus reducing its service performance and service life.

The service performance of asphalt pavement is closely associated with environmental factors. Due to the diversity and complexity of salt erosion environments, asphalt pavements suffer from multiple forms of damage under salt erosion environments. In order to further explore the salt erosion mechanism, multi-scale investigations under multi-factor coupling conditions have been conducted on asphalt materials.

**Figure 2:**
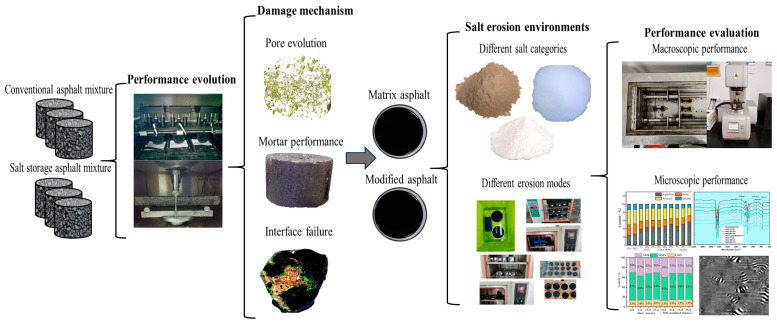
Flow chart of this research [4,6,9,10,11,12,13].

For extending the service life and reducing the maintenance costs of asphalt pavements, it is essential to reveal the damage mechanism of asphalt materials under salt erosion environments and improve the durability of asphalt pavements in salt-rich regions. There has been extensive research on the performance deterioration mechanism of asphalt materials under salt erosion conditions; however a systematic summary of salt erosion damage and the damage mechanism on asphalt materials is deficient. This article reviews the damage evolution characteristics of asphalt materials from a multi-scale perspective under salt erosion environments. The performance evolution of asphalt mixtures under salt erosion environments is summarized. Meanwhile, the damage mechanism of asphalt mixtures under salt erosion environments is discussed from three perspectives: pore properties, adhesion properties, and mortar properties. In addition, the damage characteristics of different asphalt binders under the erosion of various salt categories and various erosion conditions is discussed. Finally, the salt erosion mechanism for asphalt binders based on the microscopic scale is summarized. The flow chart is shown in Figure 2.

## 2. Damage Characteristics of Asphalt Mixture Under Salt Erosion Environments

The performance damage characteristics of asphalt mixtures under salt erosion environments have been extensively studied by domestic and foreign scholars. This includes multi-scale studies from macroscopic to microscopic and high-throughput tests from destructive to nondestructive, together with the effects of solution concentration fields [14] and external temperature fields [8] on the asphalt mixture’s performance. In addition, the pavement performance of asphalt mixtures containing salt storage additives has received wide attention due to the common international application of self-melting snow asphalt pavements [15]. Hence, the performance of both conventional asphalt mixtures and salt storage asphalt mixtures in salt erosion environments deserves to be scrutinized.

### 2.1. Performance Evolution of Asphalt Mixture Under Salt Erosion Environments

**Figure 3:**
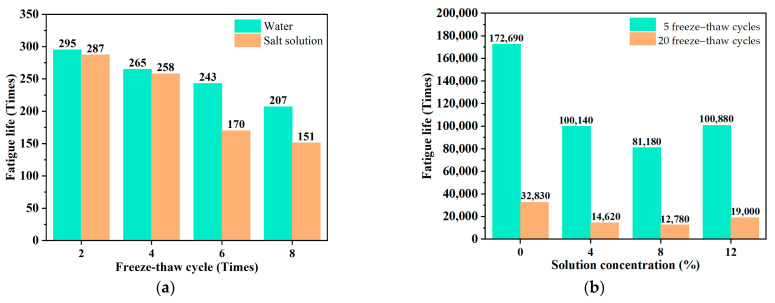
Fatigue life of asphalt mixture under salt freeze–thaw cycles: (**a**) splitting fatigue [8] and (**b**) four-point bending [17].

For the conventional asphalt mixture, the high- and low-temperature performance as well as fatigue performance decreased significantly under salt freeze–thaw cycle conditions. Feng et al. [16] investigated the influence of freeze–thaw cycles and salt on asphalt mixtures. The test results indicate that salt plays a significant role in the low-temperature performance of asphalt binders. When the salt content exceeds 3%, there is a certain degree of increase in the weight loss ratio of asphalt, accompanied by a rapid decline in its deformation capability. Wang et al. [14] employed the creep test and trabecular bending test to analyze the high- and low-temperature performance of an asphalt mixture under salt freeze–thaw cycles, and concluded that the high- and low-temperature performance of the asphalt mixture decreased due to the freezing expansion force of water and crystallization pressure of salt under the freezing process. Wu et al. [8] analyzed the influence of salt freeze–thaw cycles on the splitting fatigue life of asphalt mixtures and concluded that the fatigue life of asphalt mixtures decreased significantly due to the intrusion and crystallization of salt solution, which accelerated the stripping of asphalt from the aggregate surface. The variation in asphalt fatigue life with salt freeze–thaw cycles is shown in Figure 3a. Zhou et al. [17] investigated the influence of salt solution concentration on the four-point bending life of asphalt mixtures, and the result can be seen from Figure 3b. From Figure 3b, it can be seen that the fatigue life decreased after salt freeze–thaw cycles, and 8 wt% salt solution exerted the most significant impact on the asphalt mixture fatigue life. This is because the existence of salt can inhibit the freeze–swelling effect of moisture, which leads to a decrease in the freezing expansion pressure of the salt solution [14,17]. Furthermore, it is worth mentioning that some studies found that the indirect tensile strength of asphalt mixtures under high salt concentrations or low-temperature freeze–thaw conditions was worse than that under dry conditions, but better than that under water freeze–thaw conditions, and this phenomenon may be due to the presence of salt, which shortened the contact time between the specimen and the frozen moisture [18,19]. Under saline immersion conditions, the high- and low-temperature performance as well as moisture stability of asphalt mixtures were damaged dramatically, and the damage increased with the salt concentration [20]. Meanwhile, the indirect tensile strength and fatigue life of asphalt mixtures can be reduced due to chemical erosion, interfacial erosion, and the crystallization pressure of salt [21]. Feng et al. [16] suggested that due to the evaporation of seawater, salt crystals would mix with the asphalt mortar, thus changing the continuity of the material. Moreover, Xiong et al. [9] indicated that the void volume of asphalt can be expanded by salt crystallization under saline immersion and saline dry–wet cycle conditions. It is also interesting to note that immersion erosion in high-concentration salt solution can mitigate the strength reduction rate of the asphalt mixture due to the salt crystals filling the small voids. Furthermore, in order to accelerate the salt erosion on asphalt mixtures, dynamic salt solution scouring experiments were performed by Xiong et al. [22], and it was concluded that the scouring action can promote the stripping of asphalt from the aggregate and result in the loss of fine aggregate, and salt crystallization in asphalt mixture voids and cracks was the main cause of salt damage on the asphalt mixture. It can be concluded from the above that the high- and low-temperature, fatigue, and tensile properties all suffered significant damage under a salt erosion environment. Asphalt material deterioration, asphalt–aggregate interface damage, and pore variation are the major reasons for the performance degradation of asphalt mixtures.

For the fatigue performance of asphalt materials under salting effects, extensive research [23,24,25,26] has been conducted systematically, revealing that alkaline salts (e.g., Na_2_CO_3_) have the most severe negative impact on fatigue life, particularly when coupled with aging processes like PAV aging. Neutral salts such as NaCl and Na_2_SO_4_ also degrade fatigue resistance, with sulfate environments exacerbating healing inhibition. Conventional 70# asphalt shows the poorest durability under salt–aging interactions, while SBS-modified asphalt exhibits better salting resistance but limited advantages at high strains or alkaline conditions, likely due to modifier degradation. Aging conditions (especially PAV aging) emerge as critical factors reducing fatigue life and self-healing capacity, with strain levels demonstrating variable effects—smaller strains show temporary benefits under aging, while larger strains consistently impair durability across all conditions. Predictive models confirm that salt–aging couplings drastically shorten fatigue life, with alkaline salts causing the greatest reductions.

**Figure 4:**
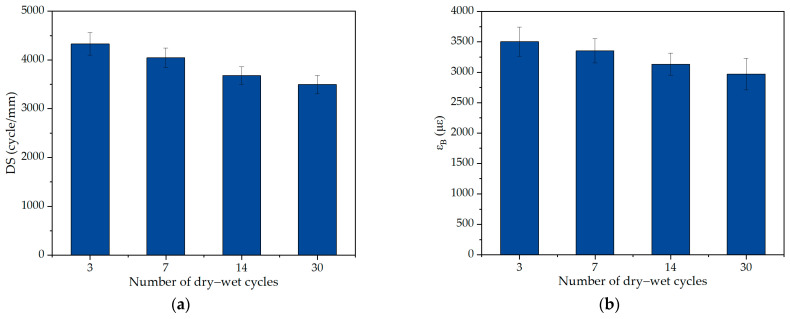
High- and low-temperature performance under salt erosion environments [28]: (**a**) dynamic stability and (**b**) trabecular bending test result.

For salt storage asphalt mixtures, the addition of salt storage additives can effectively improve the frost protection effect of the pavement, but it can significantly decrease the pavement performance of asphalt pavement [27]. The high- and low-temperature performance of asphalt mixtures evaluated by the dynamic stability and trabecular bending tests under salt erosion environments is described in Figure 4 [28]. In addition, from the chloride ion migration characteristics in salt storage asphalt mixtures, it can be obtained that a high immersion temperature and high salt storage filler content can promote the emigration of chloride ions, and the emigration of chloride ions can lead to the deposition of salt storage additives in the asphalt mixture [29]. In addition, the dissolution of salt storage additives in the deicing process can result in an increase in the porosity of the asphalt mixture, which deteriorated its freeze–thaw resistance [30]. Furthermore, a mutually reinforcing relationship was formed between freeze–thaw damage and salt storage additive dissolution, and the freeze–thaw process resulted in an increase in the porosity of the asphalt mixture, thus accelerating the dissolution of salt storage additives; meanwhile, the dissolution of salt storage additives can increase the number of pores and void content, which aggravated the freeze–thaw damage of the asphalt mixture [6]. For the purpose of improving the pavement performance of salt storage asphalt mixtures, Zhou [31] suggested that replacing lignin fibers with polyester fibers can enhance the stripping resistance of salt storage asphalt mixtures. In addition, Wang et al. [32] self-developed a kind of salt storage aggregate, and indicated that this salt storage aggregate possessed significant angularity and well-developed pores, which can improve the high- and low-temperature performance of asphalt mixtures. It can be seen that the pavement performance of salt storage asphalt mixtures decreased significantly, and the porosity variation is the main reason for the performance deterioration of salt storage asphalt mixtures.

After salt erosion, the mechanical performances of both conventional asphalt mixtures and salt storage asphalt mixtures decreased significantly. The reasons for the damage of asphalt mixtures in salt erosion environments include pore variation, failure of the asphalt–aggregate interface, and deterioration of asphalt mortar performance. The erosion mechanisms of salt on asphalt mixtures are as follows: Firstly, pore variation within the asphalt mixture occurs due to salt crystallization pressure and the dissolution of salt storage additives. Secondly, adhesion failure arises from the penetration of salt into the asphalt–aggregate interface. Thirdly, there is a deterioration in the performance of asphalt mortars. Consequently, the following section discusses the evolution of pore properties, asphalt–aggregate interface properties, and asphalt mortar properties in salt erosion environments, providing a basis for understanding the damage mechanisms of asphalt mixtures.

### 2.2. Discussion of the Pore Evolution Under Salt Erosion Environments

**Figure 5:**
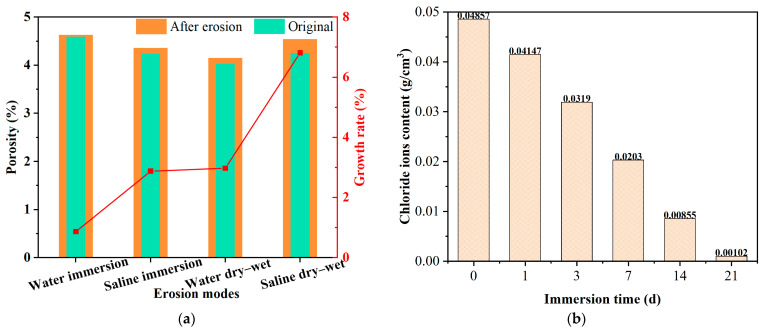
Pore variation in asphalt mixture and dissolution process of salt storage additive: (**a**) pore variation [9] and (**b**) dissolution process of salt storage additive [29].

Expansion of and increase in pores are the main reasons for salt damage on asphalt mixtures. Xiong et al. [9] employed the CT test to analyze the pore evolution of an asphalt mixture under saline immersion and salt dry–wet cycle conditions, and concluded that the crystallization pressure generated by salt in the open pores could accelerate the increase in open pore volume, which deteriorated the performance of the asphalt mixture. Zhang et al. [33] explored the pore characteristics of asphalt mixtures under salt dry–wet cycle conditions through Industrial Computer Tomography Testing, and obtained that the parameters such as total pore volume, average pore volume, and equivalent pore diameter of asphalt mixtures were significantly increased due to the crystallization expansion of salt. The characteristics of pore variation under different erosion modes are shown in Figure 5a. It can be seen from Figure 5a that the growth rate of pores is highest under saline dry–wet conditions. Wu et al. [6] used the CT test and mercury intrusion porosimetry test to investigate the pore evolution of salt storage asphalt mixtures, and indicated that the totality of pores was increased significantly due to the dissolution of salt storage additives after salt freeze–thaw cycles. Cao et al. [30] likewise suggested that the dissolution of salt storage additives can increase the number of pores, which exacerbates the freeze–thaw damage to the asphalt mixture. The variation in chloride ion content with immersion time is shown in Figure 5b. As can be seen from the result, the chloride ions in the asphalt mixture are almost eliminated after 21 days of immersion.

**Figure 6:**
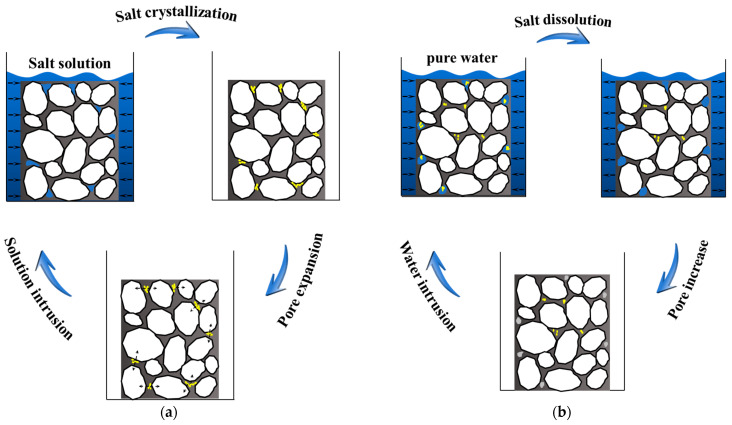
Pore evolution characteristics of asphalt mixture under salt erosion conditions: (**a**) conventional asphalt mixture and (**b**) salt storage asphalt mixture.

Figure 6 describes the pore evolution of the conventional asphalt mixture and salt storage asphalt mixture in salt-erosion environments. For the conventional asphalt mixture as shown in Figure 6a, the salt solution penetrated the asphalt mixture first, and then the crystallization of salt occurred during the freezing process or drying process; subsequently, the pores in the asphalt mixture were expanded due to the crystallization pressure of salt, which resulted in the performance deterioration of the asphalt mixture. For the salt storage asphalt mixture as shown in Figure 6b, the salt storage additive was dissolved in the wet condition, and then new pores were generated due to the dissolution of salt storage additive; subsequently, the porosity was increased during the erosion process, which degraded the performance of the salt storage asphalt mixture. In general, salt damage to asphalt mixtures is mainly attributed to pore expansion and enlargement. The crystallization pressure of salt within open pores accelerates the growth of pore volume, thereby deteriorating the performance of the mixture. Meanwhile, due to salt expansion, parameters such as total pore volume, average pore volume, and equivalent pore diameter increase significantly, exacerbating the damage.

### 2.3. Discussion of Asphalt–Aggregate Adhesion Property Under Salt Erosion Environments

**Figure 7:**
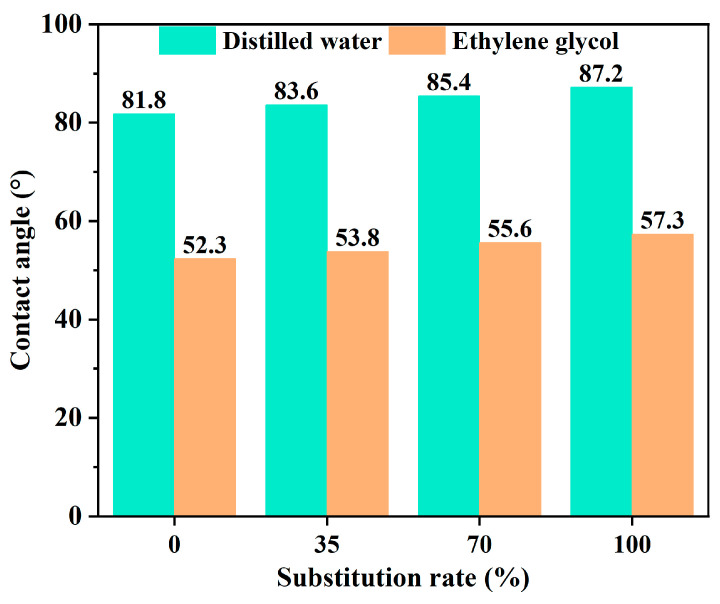
Contact angle test results of salt storage asphalt mastic [41].

Asphalt–aggregate adhesion failure is likewise one of the major reasons for the damage of asphalt mixtures in salt erosion environments. The asphalt–aggregate adhesion property deteriorated significantly after salt erosion. It has been obtained that the erosion effect of sulfate on the asphalt–aggregate interface was stronger than that of chloride salt, and the higher the solution concentration, the stronger the erosion effect [34]. From the perspective of energy, it can be obtained that the surface energy of the asphalt binder at room temperature is far lower than that of the salt solution, which leads to the coverage of the aggregate surface with more salt solution [35]. In salt erosion environments, the salt solution can not only weaken the bond strength of the asphalt binder, but also change the surface tension of the asphalt molecules. In addition, an unstable adsorption layer between the asphalt and aggregate can be generated due to the salt erosion, which reduces the asphalt–aggregate adhesion performance. In addition, the chloride and sulfate ions in the salt solution can absorb better into the aggregate, which results in easier penetration of the salt solution into the asphalt–aggregate interface [36]. It has also been shown that salt ions were stronger in polarity than asphalt, so the aggregates, with higher surface energy, would adsorb more polar salt ions to accomplish a reduction in surface energy, which resulted in a weaker affinity of aggregate with asphalt compared to salt ions [17]. Furthermore, the salt solution can accelerate the dissolution of the polar components in asphalt, reducing the polar component content and the surface free energy of asphalt, which weakens the adhesion between the asphalt and aggregate [37,38,39,40]. For the salt storage additive asphalt binder, Xu et al. [41] suggested that the presence of salt storage additives can degrade the affinity between the asphalt and aggregate. The contact angle test results of salt storage asphalt mastic are shown in Figure 7. As can be seen from Figure 7, the contact angle of salt storage asphalt mastic rises continuously with the increase in the replacement of mineral fillers by salt storage additives. The reason might be that the salt storage additive diminished the asphalt activity, which resulted in poor wettability of the asphalt binder.

In summary, the asphalt–aggregate adhesion property decreased after salt erosion, which significantly influenced the performance of the asphalt mixture under salt erosion environments. In addition, the damage of salt on asphalt–aggregate adhesion mainly includes damage to the asphalt binder and the impact on the aggregate surface. Firstly, salt solutions can decrease the cohesion of the asphalt binder and reduce its surface free energy, which deteriorates the asphalt–aggregate adhesion property. Then, the affinity between the aggregate surface and the salt ions is stronger than that with the asphalt molecules, which results in an easier access to the asphalt–aggregate interface for salt solutions compared with fresh water, thereby accelerating the stripping of the asphalt.

### 2.4. Discussion of Asphalt Mortar Property Under Salt Erosion Environments

According to modern colloid theory, asphalt mortar is the dispersion medium in the multi-phase system of asphalt mixtures [42]. Although the proportion of asphalt mortar is less than that of coarse aggregates in asphalt mixtures, it provides an important contribution to the internal bonding of asphalt mixtures [43]. Asphalt mortar can significantly influence the viscoelastic mechanical behavior of asphalt mixtures; meanwhile, it is the most vulnerable location for cracking in asphalt mixtures [44]. In addition, asphalt mortar failure is one of the major reasons for the salt erosion damage of asphalt mixtures. Hence, it is essential to analyze the damage characteristics of asphalt mortar in salt erosion environments.

At present, the damage characteristics of asphalt mortar under salt freeze–thaw cycle conditions have mainly been analyzed. It has been pointed out that during the process of salt freeze–thaw cycles, the freeze–swelling effect can expand the pores in asphalt mortar, which facilitates the penetration of salt solutions into the asphalt–aggregate interface. Subsequently, the salt solution can crystallize under freezing conditions to generate crystal expansion effects, which can destroy the structure of the asphalt mortar [4]. Fen et al. [45] also demonstrated that the crystallization of salt at low temperature could generate expansion pressure, and the formed crystals were prone to puncturing the asphalt film, which resulted in damage to asphalt mortar’s internal structure. More than that, the evaporation of moisture in the salt solution prompted salt crystallization precipitation, leading to the formation of physical adulteration between salt and asphalt mortar, which changed the continuity of asphalt mortar. Furthermore, asphalt and salt that possess different shrinkage coefficients may generate interfacial adhesion failure with variation in temperature [16]. Cui et al. [3] indicated that salt freeze–thaw cycles significantly deteriorate the axial deformation resistance of asphalt mortar. The main reasons were that the asphalt and the aggregate suffered from uncoordinated deformation during the variation in temperature, and when subjected to external forces, uncoordinated forces occurred within the asphalt mortar, which consisted of non-homogeneous materials. In summary, salt freeze–thaw cycles can significantly reduce the axial deformation resistance of asphalt mortar and generate uncoordinated forces within it, severely affecting the performance of asphalt mixtures in salt erosion environments. This is because during the process of salt freeze–thaw cycles, the freeze–swelling effect expands the pores in asphalt mortar, allowing salt solutions to penetrate the asphalt–aggregate interface and generate crystal expansion effects that destroy the structure of asphalt mortar. Additionally, the evaporation of moisture in salt solutions prompts salt crystallization precipitation, leading to physical adulteration between salt and asphalt mortar and altering the continuity of asphalt mortar. Meanwhile, due to the different shrinkage coefficients of asphalt and salt, interfacial adhesion failure can easily occur with temperature variations.

From the above studies, it can be seen that the main reasons for the damage of asphalt mortar under salt freeze–thaw cycles include the following three factors: firstly, the erosion of the asphalt–aggregate interface by water freezing expansion and salt crystalline swelling; secondly, the penetration of salts into the asphalt mortar, which deteriorates the continuity of the asphalt mortar; and thirdly, the salt, asphalt, and aggregate have different shrinkage coefficients, which results in interfacial failure and uncoordinated deformation during the variation in temperature. The low-temperature and mechanical properties of asphalt mortar under salt freeze–thaw cycles are mainly analyzed currently, and there exist certain shortages in the research on the damage evolution of asphalt mortar under different salt erosion conditions.

## 3. Performance Degradation for Asphalt Binder in Salt Erosion Environments

The performance of asphalt binders can be deteriorated under salt erosion environments, which significantly influences the asphalt–aggregate interface property and the asphalt mortar property, thereby influencing the performance of asphalt mixtures. In addition, the asphalt binder properties exhibit significant variability in different salt erosion environments. Hence, the performance evolution of different asphalt binders under the erosion of various salt categories and various erosion conditions are discussed in the next section.

### 3.1. Asphalt Binder Performance Under the Erosion of Various Salt Categories

Asphalt pavement in coastal areas suffers mainly from the erosive effects of sea salt. Sea salt contains a variety of salts, of which around 80% are chloride salts and over 10% are sulfate salts [46,47]. In saline soil regions, the salt ions that erode the asphalt pavement are extremely sophisticated, involving sulfate, chloride, and carbonate [48,49,50,51]. Furthermore, calcium chloride and sodium chloride, as traditional deicing salts, are commonly applied to deal with snow-covered pavements [52]. In recent years, acetate-based deicer has been widely used in road deicing projects due to its lower freezing point and superior deicing efficiency [7]. As a result, asphalt pavements in different regions are subject to various types of salt erosion. It is therefore necessary to discuss the influence of salt category on the performance of asphalt materials. Current research on the impact of salt category on the performance of asphalt materials primarily focuses on sulfates, chlorides, carbonates, and organic salts.

For chloride salt, Zhang et al. [1] utilized sodium chloride (NaCl) to immerse the asphalt binder, and the results indicated that salt erosion, along with a decrease in temperature, can lead to an increase in the complex modulus and rutting factor of the asphalt. This was mainly attributed to the increased hard component content of the asphalt binder after immersion in deicing salt solution; in addition, the salt ions can penetrate the asphalt binder, and then enhance the shear deformation resistance of the binder. Zhang et al. [12] conducted immersion erosion tests on the asphalt binder in sodium chloride (NaCl) and calcium chloride (CaCl_2_) solutions, respectively, and found that the complex modulus of the asphalt binder increased with salt erosion but decreased with an increase in temperature. Additionally, the phase angle decreased with salt erosion while increasing with rising temperatures. This indicated that chloride erosion can harden the asphalt while increasing its elastic content. In addition, the degradation of binder performance in sodium chloride solution was greater than that in calcium chloride solution. The reason was that asphalt binder, as an acidic material, seemed more susceptible to being eroded by weakly alkaline sodium chloride solution compared to the acidic calcium chloride solution [53].

With regard to sulfate, the thioethers and mercaptan compounds in asphalt experienced oxidative condensation reactions to create sulfoxide and disulfide compounds in sulfuric acid (H_2_SO_4_) solutions, which resulted in an increase in the asphaltene ratio. Subsequently, the sea salt erosion condition was simulated by using a mixture of NaCl and sodium sulfate (Na_2_SO_4_) solutions, and the results indicated that the sea salt erosion environment can lead to a decrease in phase angle, and an increase in complex modulus [11,54]. Zhou et al. [34] showed that Na_2_SO_4_ exerted a greater erosive action on the asphalt binder than NaCl due to the stronger erosive action of sulfate. This is attributed to the fact that sulfates deteriorate the plasticity of the asphalt binder, and sodium ions react with the light components of the asphalt binder, resulting in a decrease in the proportion of those light components.

As to carbonate, Busang et al. [2]. immersed the matrix asphalt binder into sodium bicarbonate (NaHCO_3_) solution and pointed out that the shear strain of the asphalt binder increased, and the creep recovery rate decreased after saline immersion. In addition, it has been observed that water can separate out a fraction of the organic components in the asphalt binder [55]. Li et al. [56] found that the complex modulus of asphalt materials soaked in sodium carbonate (Na_2_CO_3_) solution decreased with increasing temperature, while the opposite trend was observed for the phase angle. Additionally, they discovered that alkaline salt solutions, such as Na_2_CO_3_ solution, can promote the precipitation of water onto the organic components of the asphalt binder. This allows for the reaction of some light components in the asphalt binder, such as carboxylic acids, with Na_2_CO_3_ solution to form water-soluble soap compounds, thereby reducing the proportion of these light components within the asphalt binder.

Furthermore, in respect of organic salt, Xiao et al. [57] immersed the asphalt binder in acetate-based deicer (CH_3_COONa) solution. The results demonstrated that the acetate-based deicer significantly reduced the viscosity of the asphalt binder, with the trend of decreasing viscosity becoming more pronounced as both the solution concentration and immersion time increased. This indicated that CH_3_COONa solution can cause the softening of the asphalt binder. Qiao et al. [58] incorporated chloride, sulfate, and acetate into the asphalt binder, respectively, to accelerate salt erosion, and then found that the incorporation of chloride and sulfate could harden the asphalt binder; however, the incorporation of acetate could lead to softening of the asphalt binder.

With the exception of the categories of salts summarized above, Mafilon (MFL), as the major salt storage additive for self-melting snow asphalt pavements, can also have a negative impact on the serviceability of asphalt pavements [59]. Self-melting snow asphalt pavement has been widely applied in many countries around the world due to its advantages such as easy construction [60]. Guo et al. [28] found that the addition of MFL increased the consistency and viscosity of the asphalt binder and decreased the penetration. This indicates that the addition of MLF hardens the asphalt binder, which may be due to the dissolution and ionization of light components such as carboxylic acids and phenols in the asphalt binder during the salt erosion process. In addition, the chloride ions in MFL can accelerate the aging of the asphalt binder.

**Figure 8:**
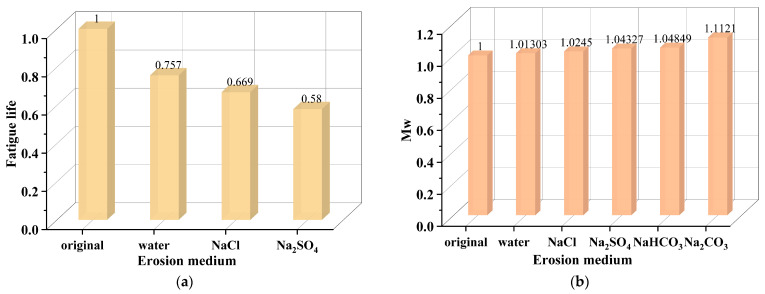
The normalized degree of impact of salt category evaluated by fatigue life and Mw: (**a**) fatigue life [61]; (**b**) Mw [62].

In conclusion, the asphalt binder exhibits significant performance variability under the erosion of different salt categories. Among them, Na_2_SO_4_ has a greater erosive effect than NaCl, and NaCl is more aggressive than CaCl_2_. In addition, acetate-based deicer can result in softening of the asphalt binder. The influence of salt category on salt erosion damage to the asphalt binder is shown in Table 1. Furthermore, in order to visualize the effect of salt category on the asphalt binder properties, the normalized degree of the impact of salt category on the matrix asphalt binder evaluated by fatigue life [61] and weight-average molecular weight (Mw) [62] under saline immersion conditions is calculated using Equation (1) and described in Figure 8. It can be concluded from Figure 8 that Na_2_SO_4_ has a greater impact on asphalt binder fatigue life than NaCl, and Na_2_CO_3_ shows the strongest influence on the Mw of the asphalt binder.

DI = EV/OV(1)

where DI represents the degree of impact of salt erosion on asphalt material performance, EV represents the erosion value, which is the indicator value after salt erosion, and OV represents the original value, which is the indicator value before salt erosion.

### 3.2. Influence of Salt Erosion Mode on the Asphalt Binder Performance

**Figure 9:**
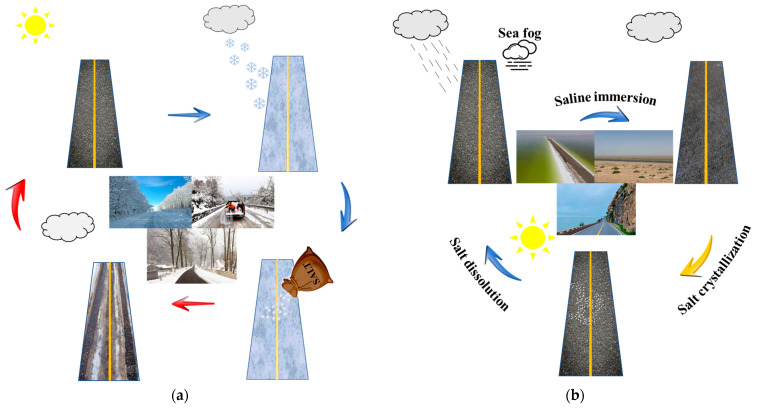
(**a**) Salt freeze–thaw erosion environment; (**b**) salt dry–wet erosion environment.

Asphalt pavement in the seasonal frozen region is exposed to salt erosion environments all year round due to pavement deicing. Almost half of China is situated in a seasonal frozen region, whose climate is characterized by high temperatures in summer accompanied by rainfall, cold winters with snowfall, and a large temperature range between day and night [63,64]. Hence, asphalt pavement in the seasonal freeze region is extremely susceptible to coupled damage from salt and freeze–thaw cycles, as described in Figure 9a. Under salt freeze–thaw cycles, asphalt pavement is prone to stripping and cracking [65]. Asphalt pavement is also subject to the erosion of seawater throughout the year in coastal areas. Under the coupling effect of high temperature and high humidity, the salt in seawater is converted from the dissolved state to the crystalline state, and then from the crystalline state to the dissolved state, which is called the salt dry–wet cycle, as shown in Figure 9b. Furthermore, the mineral powder in self-melting snow asphalt mixtures is usually replaced by salt storage additives [10,66]. The damage of salt as an internal dopant on the asphalt binder should also be considered. In conclusion, asphalt pavements are subjected to the damage of various salt-erosion environments, and it is necessary to explore the damage mechanisms of asphalt materials under different salt erosion modes.

The presence of salt can inflict additional damage to the asphalt binder under the coupling action of salt and freeze–thaw cycles. It has been shown that salt crystals can pierce the binder film and penetrate into the asphalt binder during freeze–thaw cycles, which leads to an increase in temperature sensitivity and a decrease in the high- and low-temperature performance of the asphalt binder [67]. Li et al. [68] likewise showed that the snowmelt remaining on the asphalt binder surface can penetrate it in the form of ionic crystals due to the incompatibility between snowmelt and the asphalt binder. Meanwhile, salt freeze–thaw cycles can destroy the three-dimensional grid structure of the SBS modifier. This weakened the adsorption of the modifier to the asphaltene, thus leading to an increase in the proportion of asphaltene. In addition, salt freeze–thaw cycles can result in more severe damage to SBS modifiers compared to water freeze–thaw cycles [13,69,70,71]. In summary, salt crystals can penetrate the asphalt binder, and the grid structure of the polymer modifier can be destroyed under the coupling effect of salt and freeze–thaw cycles.

Under salt dry–wet cycle and freeze–thaw cycle conditions, the salt erosion effect can promote the migration of asphaltene molecules to the binder–saline interface, thus resulting in a strong structural film at the interface [72]. In addition, the macroscopic properties of asphalt binders damaged by salt dry–wet cycles were similar to those caused by salt freeze–thaw cycles; both erosion modes improved the high-temperature performance and degraded the low-temperature performance of the asphalt binder. However, the microscopic morphology as well as the chemical compositions of the asphalt binder changed more significantly under salt freeze–thaw cycles than under salt dry–wet cycles [11,72]. It can be concluded from above that the erosion caused by the salt freeze–thaw cycles and salt dry–wet cycles was analogous, and more severe damage was caused to the asphalt binder by salt freeze–thaw cycles.

Self-melting snow asphalt pavement achieves the effect of snow elimination by releasing the salt storage additives inside the asphalt binder. However, the incorporation of salt storage additives exerts a certain negative impact on the performance of asphalt materials. Qiao et al. [58] found that the salt incorporated into the asphalt binder could absorb the light components, which led to a decrease in the percentage of light components. Moreover, the salt inside the asphalt binder can impose a negative influence on the continuous state of binder. Guo et al. [28] showed that the adhesion between the asphalt binder and salt storage additive was worse compared to conventional mineral powders, and the salt storage additives inside the asphalt binder could disrupt its continuity, which deteriorated the low-temperature cracking resistance of the asphalt binder.

**Figure 10:**
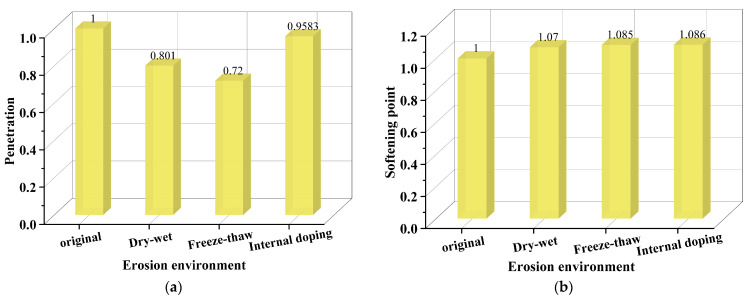
The normalized degree of impact of salt erosion environment evaluated by penetration and softening point: (**a**) penetration; (**b**) softening point [55,68].

In addition, for describing the impact of salt erosion environments on the asphalt binder more intuitively, the degree of impact of salt erosion environments on the matrix asphalt binder evaluated by the penetration and softening point at an approximate salt erosion time is calculated using Equation (1) and shown in Figure 10. From the result, salt freeze–thaw cycles exerted the heaviest impact on the penetration of the asphalt binder. In addition, salt storage additives had the most significant influence on the softening point of the asphalt binder. 

In summary, the damage characteristic of the asphalt binder is different in diverse salt erosion environments. The damage of the asphalt binder under salt freeze–thaw cycles and salt dry–wet cycles is mainly due to salt crystallization under the action of temperature and moisture, which can pierce the asphalt binder film and penetrate the asphalt binder. Meanwhile, the damage characteristic of the asphalt binder by salt dry–wet cycles is similar to that by salt freeze–thaw cycles; however, the erosion effect of salt dry–wet cycles is weaker than salt freeze–thaw cycles. In addition, when salt acts inside the asphalt binder in the form of an internal dopant, the main reason for its performance degradation is that the salt inside the asphalt binder disrupts the continuous state of the asphalt binder molecules.

### 3.3. Salt Erosion Resistance of Different Asphalt Binders

In recent years, the performance of different types of asphalt binders has been investigated in salt erosion environments by researchers. Matrix asphalt binder is widely used due to its low cost and construction convenience, and it also functions as the basis of modified asphalt binders; hence, it is essential to investigate the damage mechanism of the matrix asphalt binder in salt erosion environments. In addition, SBS is one of the most commonly used modifiers currently, and it can decrease the temperature sensitivity and enhance the high-temperature property of asphalt binders [73,74]. In recent years, rubber powder-modified asphalt binder has gained wide attention; the application of rubber powder can not only protect the environment, but also improve the high- and low-temperature performance as well as the fatigue resistance of asphalt binders [75,76]. The following section summarizes the performance evolution characteristic of the matrix asphalt binder, SBS-modified asphalt binder, rubber powder-modified asphalt binder, and other categories of asphalt binders under salt erosion environments.

Cui et al. [63] showed that salt erosion deteriorated the dispersion of asphaltenes in the matrix asphalt binder and degraded the low-temperature cracking resistance of the matrix asphalt binder. Fu et al. [77] indicated that the effect of snowmelt can deteriorate the low-temperature performance of the matrix asphalt binder, but can improve its high-temperature performance. Meanwhile, salt erosion can increase the hardness and viscosity of the matrix asphalt binder and weaken its liquidity [61]. Zhang et al. [12]. found that salt erosion contributed to an increase in the complex modulus and the deformation resistance of the matrix asphalt binder. This indicated that the matrix asphalt binder becomes hardened under the effect of salt erosion. In addition, the evolution of the chemical compositions in the matrix asphalt binder tends to be comparable to that of the aging test regardless of the salt erosion modes [72]. In conclusion, it can be seen that the performance evolution of the matrix asphalt binder in salt erosion environments is mainly characterized by the improvement in high-temperature stability and deterioration of low-temperature cracking resistance; meanwhile, this is accompanied by an increase in hardness and viscosity.

There exist studies pointing out that the high-temperature performance and elastic recovery performance of SBS-modified asphalt binder were improved under saline immersion conditions, but the low-temperature performance was reduced [11,54]. However, Li et al. [78] showed that both the high-temperature and low-temperature properties of SBS-modified asphalt binder were deteriorated under salt freeze–thaw cycles. Cui et al. [65] found out that SBS-modified asphalt binder can become softened due to the emulsification of sodium ions and the erosion of chloride ions. In addition, it has been shown that the rutting resistance of SBS-modified asphalt binder is weakened under salt freeze–thaw cycles because of the destruction of the three-dimensional mesh structure of the SBS modifier [13]. Conversely, Zhang et al. [1] pointed out that salt erosion can increase the proportion of hard components in the asphalt binder, thus enhancing the interaction forces between asphalt molecules, which in turn improves the complex modulus and rutting resistance of the asphalt binder. Furthermore, Guo et al. [28] subjected SBS-modified asphalt binder containing salt storage additives to dry–wet cycles and found that the asphalt binder became hard and brittle under salt erosion conditions. In summary, it can be seen that the performance evolution of SBS-modified asphalt binder is controversial under salt erosion conditions. This may be because diverse salt erosion modes exert different influences on SBS-modified asphalt binder and the different sources of SBS-modified asphalt binder used by researchers.

**Figure 11:**
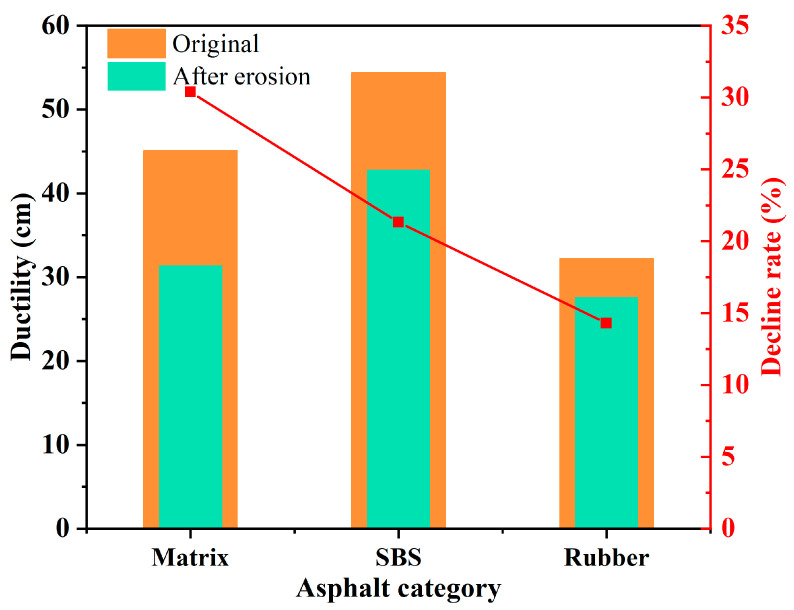
Trend in asphalt ductility under salt freeze–thaw cycles [81].

The rubber powder particles in rubber powder-modified asphalt binder would be gradually stripped from the asphalt binder during the process of salt freeze–thaw cycles, thus destroying the structure of rubber powder-modified asphalt binder [78]. Cui et al. [79] found that salt erosion deteriorated the stress relaxation capacity and low-temperature crack resistance of rubber powder-modified asphalt binder; nevertheless, the low-temperature performance of the rubber powder-modified asphalt binder was also better than that of the matrix asphalt binder. Meanwhile, the rubber particles can evenly distribute in the asphalt binder in the form of particulates and chains, which reduces the aggregation of asphaltenes; hence, the asphaltene can better disperse in the rubber power-modified asphalt binder than the matrix asphalt binder during salt erosion [63,80]. In addition, compared to the matrix asphalt binder and SBS-modified asphalt binder, rubber powder-modified asphalt binder possessed better performance stability under salt freeze–thaw cycles [69]. Therefore, rubber powder-modified asphalt binder is more suitable for asphalt pavement construction under a salt freeze–thaw environment than the matrix asphalt binder as well as SBS-modified asphalt binder. In addition, the ductility of the matrix asphalt binder, SBS-modified asphalt binder, and rubber powder-modified asphalt binder before and after salt freeze–thaw cycles, together with their decline rates, is described in Figure 11. It can be obtained from Figure 11 that rubber power-modified asphalt binder possesses the lowest decline rate of ductility. This indicates that rubber powder-modified asphalt binder has the best resistance to salt freeze–thaw cycles compared with the matrix asphalt binder and SBS-modified asphalt binder.

In the case of other categories of asphalt binders, Busang et al. [2] found that Teak (TK) fiber-modified asphalt binder was superior to the matrix asphalt binder in resistance to salt erosion. Yu et al. [82] conducted salt erosion on the matrix asphalt binder after long-term aging, and showed that salt erosion improved the high-temperature performance of the aged asphalt binder; however, the low-temperature performance as well as the fatigue resistance were deteriorated. In addition, aged asphalt binder exhibited more stable properties compared to unaged asphalt binder under salt erosion environments [12]. Meng et al. [83] showed that Trinidad Lake Asphalt (TLA)-modified asphalt binder provided better salt resistance than the matrix asphalt binder. Yin et al. [64] analyzed the performance evolution regularity of various modified asphalt binders under salt freeze–thaw cycles, and the results showed that PE can enhance the high-temperature stability of asphalt binders under salt freeze–thaw conditions, while lignin fiber and polyester fiber can significantly enhance the low-temperature crack resistance of asphalt binders. Furthermore, Zhang et al. [84] analyzed the performance of recycled asphalt binders in salt erosion environments, and then concluded that recycled asphalt binder exhibited superior salt resistance than the matrix asphalt binder due to the fact that the regenerants in recycled asphalt binder can mitigate the aging effect. In the study of modified asphalt binders, it has been discovered that the incorporation of various modifiers can significantly alter the performance characteristics of asphalt compared to matrix asphalt. These findings collectively underscore the potential of modified asphalt binders to enhance the durability and performance of asphalt pavements in salt erosion environments.

Different types of asphalt binders present significant performance variability in salt erosion environments. The high-temperature performance and complex modulus of the matrix asphalt binder are increased under salt erosion environments, but the low-temperature performance is decreased; meanwhile, the asphalt binder can become brittle and hard. In addition, there exist some controversies about the performance evolution of SBS-modified asphalt binder in salt erosion environments; this may be due to the diverse test conditions, as well as the different source of SBS-modified asphalt binder. Hence, the damage evolution mechanism of SBS-modified asphalt binder in salt erosion environments needs to be further investigated. Furthermore, the high- and low-temperature performance of rubber powder-modified asphalt binder is more excellent than the matrix asphalt binder and SBS-modified asphalt binder under salt freeze–thaw cycle conditions, especially the low-temperature performance. In general, SBS-modified asphalt binder outperforms the matrix asphalt binder in salt erosion environments, and rubber powder-modified asphalt binder should be preferred in salt freeze–thaw cycle conditions.

In addition, from an economic perspective, salt erosion, particularly under freeze–thaw cycles (e.g., NaCl or Na_2_CO_3_ exposure), significantly accelerates fatigue degradation in asphalt materials, with quantified impacts on maintenance costs and cycles. For conventional asphalt mixtures, salt freeze–thaw cycles reduce fatigue life by approximately 50% [8], shortening maintenance intervals from the standard 5 years to 2.5 years, thereby doubling annual maintenance costs. Similarly, base asphalt binders exposed to saline environments (e.g., 10% NaCl immersion) exhibit a 30–50% fatigue life reduction [33], necessitating more frequent repairs. Modified asphalts demonstrate better resistance: SBS-modified asphalt shows 30–40% fatigue life decline under salt freeze–thaw conditions, extending maintenance cycles to 3.25 years, while rubber-modified asphalt, with only 20–30% fatigue deterioration, prolongs intervals to 3.75 years. Notably, alkaline salts like Na_2_CO_3_ cause the most severe damage, degrading fatigue life by 60–80% under PAV aging [23], which could push maintenance costs for unprotected pavements to unsustainable levels. Economically, prioritizing rubber-modified or composite binders in high-salinity regions (e.g., coastal or saline soil areas) reduces long-term costs by 33–54% compared to conventional asphalt. Proactive measures—such as pore structure monitoring and chemical-resistant modifiers—are critical to mitigate salt-induced aging and optimize lifecycle budgets, particularly under multi-factor erosion (salt–UV load coupling) prevalent in harsh climates.

## 4. Evolution of Chemical Compositions and Microscopic Morphology of Asphalt Binder Under Salt Erosion Environments

In recent years, research on asphalt materials has gradually transitioned from macroscopic analysis to mesoscopic and microscopic analysis due to the continuous advancement of testing equipment. For example, Fourier Infrared Spectroscopy (FTIR), Gel Permeation Chromatography (GPC), and the Four-Component Separation Assay (SARA) can accurately characterize the evolution of the chemical compositions of asphalt materials [85,86,87,88]. In addition, Atomic Force Microscopy (AFM) is an effective technique to analyze asphalt microscopic morphology [89]. The evolution characteristics of the chemical compositions and microscopic morphology of asphalt materials in salt erosion environments can be analyzed more accurately by means of these micro-scale technologies.

### 4.1. Effect of Salt-Erosion Environments on Chemical Compositions of Asphalt Binder

FTIR tests have been widely used for asphalt materials to analyze the evolution of asphalt functional groups in salt erosion environments. Guo et al. [28] showed that the incorporation of salt storage additives did not lead to the appearance of new absorption peaks, which indicated that there existed only physical doping without chemical reaction between the salt storage additives and the asphalt binder. Qiao et al. [58] incorporated salt into the asphalt binder to substitute mineral powder, and the FTIR results indicated that the physical interaction mainly occurred between asphalt binder and salt. Xiong et al. [90] conducted an FTIR test on salt-doped asphalt mastic, and the results showed that the incorporation of salt does not create new functional groups. This indicates that the binder–salt interface is dominated by physical interaction. However, the existing research has shown that saline immersion can lead to an increase in the total absorption intensity of FTIR of the asphalt binder, and the light component of the asphalt binder can be transformed to the heavy component; hence, a partial chemical reaction occurs between the asphalt binder and salt [1]. Meng et al. [61] conducted an FTIR test on an asphalt binder immersed in salt solution, and showed that salt erosion can induce variation in the intensity of the absorption peaks; meanwhile new absorption peaks appeared, which suggested that a chemical reaction may have occurred between the salt solution and the asphalt binder. Zhang et al. [54] likewise concluded that salt solution can react chemically with the asphalt binder, and showed that after salt erosion, four new absorption peaks appeared in the FTIR test; meanwhile the area of the peaks representing aromatic and SBS modifiers was reduced. In addition, it has been obtained that the Cl element in the salt solution can substitute the H element of the C-H bond in the asphalt binder, thus forming a new C-Cl functional group [12]. In summary, it can be obtained that when salt is internally incorporated into the asphalt binder in the form of a solid, physical interaction is likely to be dominant between salt and the asphalt binder. However, when salt erodes the asphalt binder in the form of salt solution, chemical interaction may take place between salt and the asphalt binder. Some functional groups and their corresponding wavenumbers are shown in Table 2. In addition, the FTIR test results of some research are summarized in Table 3. As observed, there is an increase in the number of ${NH}_{2}^{+}$, -CH_2_-, and -CH_3_- groups within the asphalt. Additionally, the content of aromatic polycyclic compounds and long-chain cycloalkanes has also increased. The saturated fractions, aromatic hydrocarbons, and the benzene ring substitution region have been affected by the presence of salt, accelerating the aging process of the asphalt. The presence and reaction of salts in the form of salt solutions exert a significant impact on the chemical composition of asphalt and accelerate its aging process.

**Figure 12:**
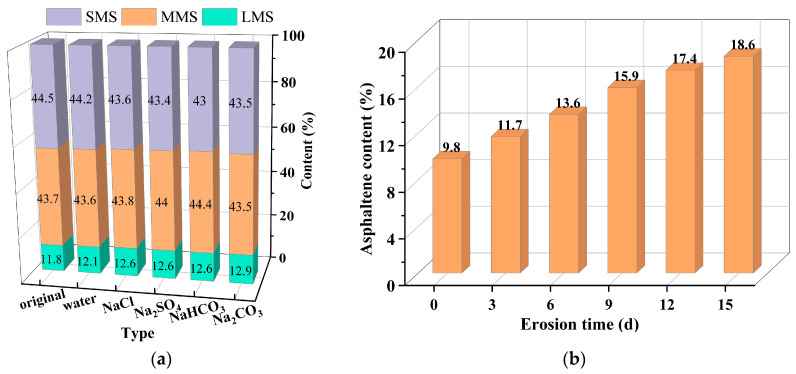
Results of GPC test and SARA test: (**a**) GPC test result [62]; (**b**) asphaltene content [72].

For other micro-indicators, Zhang et al. [94] and Meng et al. [62] conducted GPC tests and concluded that salt erosion resulted in an increase in the large molecule size (LMS) content of the asphalt material. Zhang et al. [12] carried out Energy-Dispersive Spectroscopy (EDS) for elemental analysis of the asphalt binder after saline immersion, and found that the percentage of O, S, and N elements in the asphalt binder increased after salt erosion, and the elements Na, Ca, and Cl appeared, as shown in Table 4. The increased content of S and N elements, which are mainly present in asphaltene and resin, suggests that salt erosion can result in an increase in the content of large molecules in the asphalt binder. Meanwhile, some elements from the salt solution can penetrate the asphalt binder. In addition, salt erosion can contribute to an increase in the percentage of asphaltene and a decrease in the percentage of saturated and aromatic components based on the SARA; however, no uniform conclusion has been reached on the variation in resin content [61,72,95]. Figure 12 shows the results of the GPC test and asphaltene content of the SARA test after salt erosion, respectively. Figure 12a illustrates an increasing trend in the large molecule size (LMS) component within the eroded samples, whereas the small molecule size (SMS) component exhibits an opposite trend. Simultaneously, as shown in Figure 12b, the asphaltene content increases by 90.8%. This demonstrates that, following salt erosion, the proportion of asphaltene in asphalt significantly increases, while the proportions of saturated and aromatic components show an opposite trend. This indicates that salt erosion accelerates the conversion from SMS to LMS in asphalt. The primary reason for this is that the salt erosion environment expedites the aging process of the asphalt binder. Light oxygenated compounds such as carboxylic acids and phenols in the asphalt binder dissolve and ionize to a certain extent, leading to a reduction in the content of saturated and aromatic components to varying degrees.

### 4.2. Microscopic Morphology of Asphalt Binder Under Salt Erosion Environments

**Figure 13:**
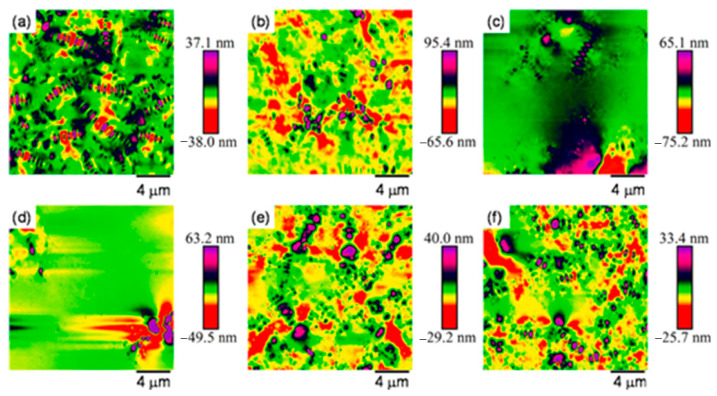
Variation in bee structures with salt solution concentration: (**a**) air, (**b**) water, (**c**) 1.5% salt solution, (**d**) 3% salt solution, (**e**) 4.5% salt solution, and (**f**) 10% salt solution [100].

AFM, as an instrument for surface analysis of materials at the nano-scale, is widely used to investigate the microscopic morphology evolution of asphalt materials [96,97]. Hence, AFM can effectively analyze the evolution regularity of asphalt binder microscopic morphology after salt erosion. The microscopic morphology of the asphalt binder under AFM is usually characterized by a “bee structure”. The formation of the bee structure is mainly related to the asphaltene and the wax component [98,99]. Long et al. [100] analyzed the surface microscopic morphology of the matrix asphalt binder immersed in different concentrations of chloride salt solution, and the results revealed that the surface roughness of the asphalt binder decreased with the increase in salt solution concentration, and the number of bee structures on the binder surface tended to decrease until disappearing and then reappearing. As illustrated in Figure 13, under normal conditions, the terrain of the base asphalt is characterized by the distribution of numerous small honeycomb-like structures. As the concentration of the salt solution increases (1.5%, 3.0%), these honeycomb-like structures gradually diminish until they completely disappear. This may be attributed to the formation of a water-soluble amorphous film on the asphalt surface by low-concentration chloride salts, which envelops the “bee structures”. The reason is that chloride and sodium ions can accelerate the movement of asphaltene molecules at the interface, leading to the formation of amorphous films and subsequently causing salt aging effects. Furthermore, the dissolution of water-soluble components and hydrophilic groups within the asphalt membrane may also contribute to the formation of these amorphous films. As the salt concentration continues to rise (4.5%, 10%), the erosive effect of the salt gradually intensifies, leading to the dissolution of the amorphous film and, consequently, the re-exposure of the bee structures. Zhang et al. [72] utilized AFM to analyze the matrix asphalt binder after salt erosion, and concluded that the surface roughness of asphalt binder decreased after salt dry–wet cycles and salt freeze–thaw cycles. In addition, after salt freeze–thaw damage, the bee structures of the matrix asphalt binder became larger and longer due to the increase in asphaltene content, and the bee structures size of SBS-modified asphalt binder became smaller but more numerous, while no bee structures appeared in rubber powder-modified asphalt binder, and there was minor difference in the surface microscopic morphology of rubber powder-modified asphalt binder before and after erosion [13,70,101].

### 4.3. Micro-Scale Damage Mechanism of Salt-Eroded Asphalt Binder

It can be obtained from the micro-scale test results that salt erosion can exert a significant influence on the chemical compositions and microscopic morphology of asphalt binders. Under salt erosion environments, the variation in the chemical compositions of the asphalt binder can be mainly characterized by a decrease in the light component proportion and an increase in the heavy component proportion and large-molecule content. The heavy component has a large molecular weight, small intermolecular distance, and strong molecular interactions [102,103,104,105]. Hence, the growth in the proportion of heavy components can increase the complex modulus and viscosity of the asphalt binder, which significantly influences the serviceability of asphalt material. In addition, there exists a correlation between the microscopic morphology of the asphalt binder and its chemical compositions [106]. The decline in the light component proportion reduces the number of dentate protrusions on the binder surface [72], which results in a decrease in the surface roughness of the asphalt binder.

**Figure 14:**
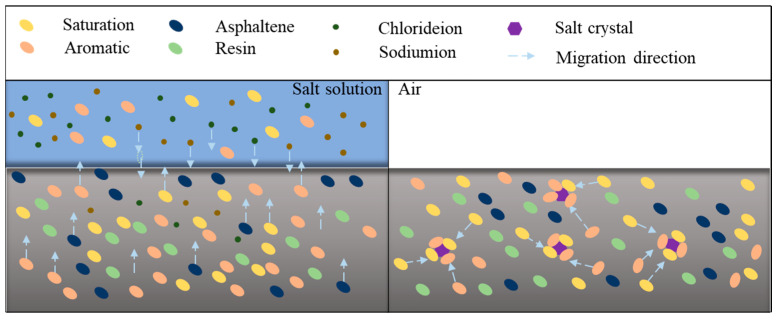
Damage mechanism of asphalt binder under external erosion (**left**) [72] and internal erosion (**right**) of sodium chloride [28].

Combined with the influence of salt erosion environments on asphalt binder viscoelastic properties as well as microscopic morphology, it can be concluded that the salt erosion on the asphalt binder can be mainly divided into physical erosion and chemical erosion, and physical erosion is the major factor. Physical erosion is primarily the migration of chemical compositions, the dissolution of polar components, and the intrusion of salt ions. Chemical erosion mainly consists of the substitution of hydrogen atoms in the asphalt binder by chloride ions, saponification reactions between components such as dissolved or ionized carboxylic acids and phenols with metal cations in salt solutions, and the conversion of light components to heavy components in the asphalt binder. Furthermore, for salt storage asphalt binders, the damage of salt to the asphalt binder is dominated by physical erosion, which includes the absorption of salt on asphalt binder light components and the destruction of binder continuity caused by salt. Figure 14 exhibits the damage mechanism of the asphalt binder under external versus internal salt erosion conditions (sodium chloride as an example).

As shown in Figure 14, under external salt erosion conditions (e.g., salt freeze–thaw cycles, salt dry–wet cycles, and saline immersion), some of the light components in the asphalt binder would dissolve or ionize, and the more polar asphaltenes would migrate toward the asphalt binder surface due to the polarization-inducing effect of salt ions. In addition, the salt ions can penetrate into the asphalt binder due to the concentration gradient effect [72]. When salt acts as an internal dopant inside the asphalt binder (e.g., salt storage asphalt binder), the salt particles can adsorb the light components of the asphalt binder, thereby reducing the proportion of light components in the asphalt binder. Meanwhile, due to the angularity of the salt particles, stress concentration may develop under the load, which destroys the continuity of the asphalt binder [28].

## 5. Conclusions and Perspectives

The presence of salt can severely damage the performance of asphalt pavements. This paper reviews the performance evolution and damage mechanism of asphalt materials under salt erosion environments based on a multi-scale perspective. The conclusions are as follows:

(1) After salt erosion, the pavement performance of both conventional asphalt mixtures and salt storage asphalt mixtures declined significantly. The primary causes of damage to asphalt mixtures in salt erosion environments are pore variation, asphalt–aggregate interface failure, and the deterioration of asphalt mortar performance. Specifically, the pores in conventional asphalt mixtures expand due to salt crystallization pressure, while new pores are generated in salt storage asphalt mixtures due to the dissolution of salt storage additives. The adhesion failure is mainly due to the cohesion reduction in the asphalt binder and the greater affinity of the aggregate with salt ions than with asphalt molecules. In addition, continuity deterioration caused by salt penetration and asphalt–aggregate interface failure are the main reasons for the deterioration of asphalt mortar performance.

(2) With regard to the salt categories, the erosion effect of SO_4_^−2^ on the asphalt binder is stronger than Cl^−^. In addition, most types of salt can increase the complex modulus, and result in the hardening of asphalt. However, acetates can reduce the viscosity of asphalt and increase its penetration, which leads to the softening of asphalt.

(3) For different salt erosion modes, the damage of the asphalt binder under salt freeze–thaw cycles and salt dry–wet cycles is mainly due to salt crystallization under the action of temperature and moisture. Meanwhile, the influence of salt dry–wet cycles on the macroscopic properties of the asphalt binder is comparable to that of salt freeze–thaw cycles, and the erosion effect of salt dry–wet cycles is weaker than that of salt freeze–thaw cycles. In addition, when the salt acts as an internal dopant in the asphalt binder, the continuity of asphalt molecules can be broken by the salt.

(4) The performance of SBS-modified asphalt is superior to matrix asphalt in salt erosion environments. Rubber powder-modified asphalt should be preferred under salt freeze–thaw cycle conditions. Furthermore, the addition of modifiers (TLA, PE, etc.) and fibers (lignin fibers, polyester fibers, etc.) can improve the salt resistance of the asphalt binder.

(5) The physical interaction between salt and asphalt primarily occurs when the salt is incorporated into the asphalt in solid form. When the asphalt is eroded by salt solutions, chemical reactions may take place between the salt and the asphalt. Meanwhile, salt erosion exerts a significant influence on the chemical compositions and microscopic morphology of the asphalt binder. In salt erosion environments, the chemical composition of the asphalt binder primarily undergoes changes manifested by a decrease in the proportion of light components, an increase in the proportion of heavy components, and an elevation in the content of large molecules. These changes lead to an increase in the complex modules and viscosity of the asphalt mixture. Furthermore, there exists a correlation between the microscopic morphology of the asphalt binder and its chemical compositions. The reduction in the proportion of light components results in a decrease in the number of dentate protrusions on the binder surface, consequently lowering the surface roughness of the asphalt binder.

Perspectives for the future:

(1) The performance of asphalt pavements is usually threatened by the coupling effects of salt, vehicle loading, and UV light concurrently during service; hence, it is necessary to analyze the coupling effects of salt erosion and multiple other factors on asphalt pavements.

(2) Molecular dynamics simulation is an effective tool to study the damage mechanism of asphalt. At present, the specific reaction mechanism between salt and asphalt is not clear; hence, conjoint analysis between laboratory tests and molecular dynamics simulation can be attempted to reveal the reaction mechanism between salt and asphalt.

(3) Asphalt mortar is an important component of asphalt mixtures. However, the damage evolution of asphalt mortar under various salts and different erosion modes is not well developed. Therefore, the salt erosion damage characteristics of asphalt mortar can be focused on in future research.

(4) Modified asphalt has become an important measure to improve the performance of asphalt pavements. The current modification of asphalt in salt erosion environments mainly focuses on a single modification. A composite modification can be considered to enhance the performance of asphalt in salt erosion environments in future research.

## Figures and Tables

**Table 1:** Influence of salt category on salt erosion damage to asphalt binder.

Salt Ion	Salt	Major Findings	Main Reason	References
Cl^−^	NaCl	Increase in complex modulus and deformation resistance, decrease in phase angle	Sodium ions reacted with light components in the asphalt binder, resulting in a decrease in the proportion of light components	[1,12,56]
	CaCl_2_	Increase in complex modulus and deformation resistance, decrease in phase angle	-	[12]
SO_4_^2−^	H_2_SO_4_	Increase in softening point and complex modulus, decrease in phase angle	Reaction in thioether and thiol compounds with sulfuric acid solutions to create sulfoxide compounds and disulfides, resulting in an increased proportion of asphaltenes	[56]
	Na_2_SO_4_	Increase in complex modules and viscosity, decrease in ductility	The plasticity of the asphalt binder was deteriorated by sulfates, and sodium ions reacted with the light components of the asphalt binder, resulting in a decrease in the proportion of light components	[56,58,61]
HCO_3_^−^/CO_3_^2−^	NaHCO_3_	Increase in shear strain and decrease in creep recovery rate	-	[2]
	Na_2_CO_3_	Increase in softening point and complex modulus, decrease in phase angle	Carboxylic acid reacted with sodium carbonate solution to form water-soluble soap compounds, resulting in a decrease in the proportion of light components	[56]
CH_3_COO^−^	CH_3_COONa	Increase in penetration, decrease in viscosity, and lead to asphalt binder softening	Acetate ions contain lipophilic groups (CH_3_-) and hydrophilic groups (COO-) that soften the asphalt binder	[57,58]
Salt additive	MFL	Increase in consistency and viscosity, decrease in penetration, and lead to asphalt binder hardening	Carboxylic acids and phenols in asphalt binder were dissolved and ionized in salt solutions, resulting in a decrease in the proportion of light components	[28]

**Table 2:** Functional groups and their corresponding wavenumbers [1,12,28,54,61,90,91,92,93].

Wavenumber (cm^−1^)	Representative
2924	Typical asymmetric stretching vibration of methylene C–H
2852	Symmetric stretching vibration of methylene C–H
1461	Scissor vibration of methylene -CH_2_-
1377	Umbrella vibration of methyl CH_3_-
812 and 868	Stretching vibrations of the benzene ring
724	Synergistic vibrations of methylene segment (CH_2_)_n_ (n ≥ 4)
747	Bending vibrations of aromatic branch chain
Near 1600	Vibration of benzene ring skeleton and hydroxyl group
1030	Stretching vibrations of the sulfoxide group (S = O)
1700	Stretching vibrations of the carbonyl group (C = O)
966~698	Benzene ring substitution area
930	Stretching vibration of C-O
840 and 780	Out-of-plane bending vibration of =CH, or stretching vibration of S-O
630	Asymmetric variable-angle vibration of ${SO}_{4}^{-2}$, or in-plane bending vibration of ${SO}_{3}^{-2}$
966	Out-of-plane bending vibration of -CH = CH-
745	Stretching vibrations of C-Cl
1133	Plan deformation of tertiary alcohol
1617	Bending vibration of ${NH}_{2}^{+}$
3467	Stretching vibration of OH^−^

**Table 3:** Summary of FTIR test results.

Indicator Variation	Erosion Modes	Explanation	References
Increased at 2924 cm^−1^, 2852 cm^−1^, 1461 cm^−1^, and 1377 cm^−1^, and enhanced at 812 cm^−1^, 868 cm^−1^, and 724 cm^−1^	Saline immersion	The increase in -CH_2_- and -CH_3_-, and the increase in the content of aromatic polycyclic compounds and long-chain cycloalkanes.	[1]
Varied at 1456 cm^−1^, 1377 cm^−1^, and 966~698 cm^−1^	Salt storage additive internal doping	The absorption peaks of saturated fractions, aromatic hydrocarbons, and the benzene ring substitution region were affected, resulting in salt aging effects.	[28,90]
New absorption peaks appeared at 930 cm^−1^, 840 cm^−1^, 780 cm^−1^, and 630 cm^−1^, and the peak areas decreased at 1377 cm^−1^ and 966 cm^−1^	Saline immersion	The salts penetrated them and reacted with the asphalt binder, and the content of aromatic and SBS modifiers was reduced.	[54]
Increased at 745 cm^−1^, 1030 cm^−1^, 1720 cm^−1^, and 1133 cm^−1^	Saline immersion	Asphalt oxidation occurred during the salt erosion, and the element Cl replaced the element H in the asphalt binder, leading to an increase in asphalt binder aging.	[12]
Affected the absorption peak near 1600 cm^−1^	Saline immersion	The chloride salt exerted a significant impact on the absorption peaks representing the aromatic fraction and the benzene ring substitution region.	[91]
New absorption peaks appeared near 1617 cm^−1^ and at 3467 cm^−1^, and peak intensity varied at 2925 cm^−1^ and 1450 cm^−1^	Saline immersion	Sodium chloride resulted in enhanced vibration or increased content of ${NH}_{2}^{+}$ groups; OH^−^ in sodium acetate reacted with acid groups in the asphalt binder; sodium sulfate led to reduced bond energy or content of -CH_2_	[61]

**Table 4:** EDS test results of asphalt binder after salt erosion [12].

Erosion Environments	Element Content (%)						
	C	O	S	N	Cl	Na	Ca
Undamaged	98.6	1.0	0.4	-	-	-	-
10% NaCl for 90d	93.2	3.2	2.0	-	0.7	0.9	-
10% CaCl_2_ for 90d	93.8	3.0	2.2	-	0.6	-	0.4

## Data Availability

Not applicable.
